# New Insights in the Immunobiology of IL-1 Family Members

**DOI:** 10.3389/fimmu.2013.00167

**Published:** 2013-07-08

**Authors:** Frank L. van de Veerdonk, Mihai G. Netea

**Affiliations:** ^1^Department of Medicine, Radboud University Nijmegen Medical Center, Nijmegen Institute for Infection, Inflammation and Immunity (N4i), Nijmegen, Netherlands

**Keywords:** interleukin-1, cytokines, ligands, review, biology

## Abstract

The interleukin-1 (IL 1) family of ligands is associated with acute and chronic inflammation, and plays an essential role in the non-specific innate response to infection. The biological properties of IL 1 family ligands are typically pro-inflammatory. The IL 1 family has 11 family members and can be categorized into subfamilies according to the length of their precursor and the length of the propiece for each precursor (Figure 1). The IL 1 subfamily consists of IL 1α, IL 1β, and IL 33, with the longest propieces of the IL 1 family. IL 18 and IL 37 belong to the IL 18 subfamily and contain smaller propieces than IL 1 and IL-33. Since IL 37 binds to the IL 18Rα chain it is part of the IL 18 subfamily, however it remains to be elucidated how the propiece of IL 37 is removed. IL 36α, β, and γ as well as IL 36 Ra belong to the IL 36 subfamily. In addition, IL 38 likely belongs to this family since it has the ability to bind to the IL 36R. The IL 36 subfamily has the shortest propiece. The one member of the IL 1 family that cannot be categorized in these subfamilies is IL 1 receptor antagonist (IL 1Ra), which has a signal peptide and is readily secreted. In the present review we will describe the biological functions of the IL-1F members and new insights in their biology.

## Introduction

**Figure 1:**
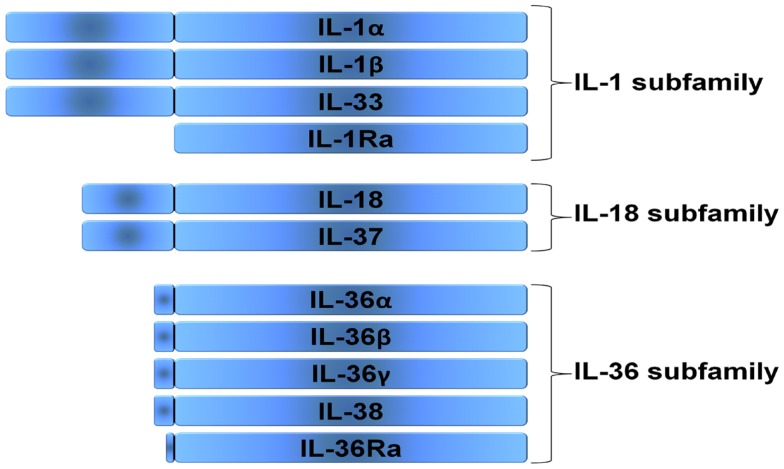
**Subfamilies according to the length of their precursor**. Three families can be distinguished in the IL-1 family, the IL-1 subfamily, the IL-18 subfamily, and the IL-36 subfamily. The IL-1 receptor antagonist (IL-1Ra) cannot be categorized in these subfamilies, since it has a signal peptide and is readily secreted.

The interleukin-1 (IL-1) family of ligands is associated with acute and chronic inflammation, and plays an essential role in the non-specific innate response to infection. The biological properties of IL-1 family ligands are typically pro-inflammatory. The IL-1 family has 11 family members and can be categorized into subfamilies according to the length of their precursor and the length of the propiece for each precursor (Figure 1). The IL-1 subfamily consists of IL-1α, IL-1β, and IL-33, with the longest propieces of the IL-1 family. IL-18 and IL-37 belong to the IL-18 subfamily and contain smaller propieces then IL-1 and IL-33. Since IL-37 binds to the IL-18Rα chain it is part of the IL-18 subfamily, however it remains to be elucidated how the propiece of IL-37 is removed. IL-36α, β, and γ as well as IL-36 Ra belong to the IL-36 subfamily. In addition, IL-38 likely belongs to this family since it has the ability to bind to the IL-36R. The IL-36 subfamily has the shortest propiece. The one member of the IL-1 family that cannot be categorized in these subfamilies is IL-1 receptor antagonist (IL-1Ra), which has a signal peptide and is readily secreted. In the present review we will describe the biological functions of the IL-1F members and new insights in their biology.

## The IL-1 Subfamily

### Interleukin-1α

IL-1α does not have a signal peptide, binds to nuclear DNA, and is released from the cell upon death after which it can bind to the IL-1R1 receptor as either an unprocessed precursor or a processed protein. Primary cells such as keratinocytes, thymic epithelium, hepatocytes, endothelial cells, fibroblasts, and the epithelial cells of mucus membranes contain constitutive levels of intracellular IL-1α precursor (Hacham et al., 2002). Furthermore, precursor IL-1α can be found on the surface of several cells, particularly on monocytes and B-lymphocytes, referred to as membrane bound IL-1α (Kurt-Jones et al., 1985). Membrane bound IL-1α is biologically active (Kaplanski et al., 1994), and its biological activities are neutralized by antibodies specific to IL-1α. Endothelial cells undergoing stress-induced apoptosis release membrane apoptotic body-like particles containing full-length IL-1α precursor and the processed mature form (Berda-Haddad et al., 2011). When injected into mice, apoptotic body-like particles containing the IL-1α precursor induce neutrophilic infiltration that can be prevented by neutralization of IL-1α (Berda-Haddad et al., 2011). Although the IL-1α precursor is biologically active, the processed form is more active. The processing of the IL-1α precursor is accomplished by calpain II, a membrane-associated, calcium-dependent cysteine protease (Miller et al., 1994), and calcium influx induces IL-1α secretion of the processed form (Gross et al., 2012).

It has been proposed that IL-1α acts as an autocrine growth factor since the intracellular regulating normal cellular differentiation, particularly in epithelial and ectodermal cells. In support of this concept, neutralizing intracellular IL-1α reduces senescence in endothelial cells (Maier et al., 1990), and constitutive IL-1α precursor can bind to HAX-1 in fibroblasts that subsequently translocates as a complex to the nucleus (Kawaguchi et al., 2006). Although these data support the concept that IL-1α can act as an autocrine growth factor, it should be noted that mice deficient in IL-1α show no defects in growth and development, including skin, fur, epithelium, and gastrointestinal function (Horai et al., 1998). However, since mice deficient in IL-1α still retain the N-terminal propiece (Werman et al., 2004) and this N-terminal propiece of IL-1α has been shown to bind HAX-1 (Yin et al., 2001) it could still be that the propiece of IL-1α is responsible for the proposed autocrine growth factor function of IL-1α.

IL-1α plays an important role in sterile inflammation. Upon necrotic cell death the IL-1α precursor is released (Carmi et al., 2009; Cohen et al., 2010) and binds to the IL-1 receptor on nearby tissue macrophages and epithelial cells (Luheshi et al., 2011; Rider et al., 2011). This will trigger a pro-inflammatory response characterized by neutrophilic influx that is followed by influx of monocytes (Rider et al., 2011). This is underlined by the observation that extracts of tumor cells induce neutrophilic inflammation, which does not occur in mice deficient in IL-1RI and that can be prevented by neutralization of IL-1α (Chen et al., 2007). Thus, IL-1α, either the unprocessed precursor or the cleaved form can be seen as an alarmin (Chan et al., 2012). Furthermore, platelets also contain IL-1α (Hawrylowicz et al., 1989), and platelet-derived IL-1α has been described to be important in brain injury in stroke models (Thornton et al., 2010) and in atherosclerosis (Gawaz et al., 2000).

In mice fed a high-fat diet, serum amyloid A protein, a marker of inflammation in atherogenesis, was markedly lower in IL-1α-deficient mice compared to wild type or IL-1β-deficient mice (Kamari et al., 2007). IL-1α-deficient mice had significantly higher levels of non-high density lipoprotein cholesterol. The beneficial effect of IL-1α deficiency was due to hematopoietic cells transferred from the bone marrow of IL-1α-deficient mice resulting in a reduction in aortic lesion size twice that observed in mice transplanted with IL-1β-deficient bone marrow cells. Therefore, IL-1α appears to play an important role in the pathogenesis of lipid-mediated atherogenesis and this may be due to an effect of membrane IL-1α.

### Interleukin-1β

**Figure 2:**
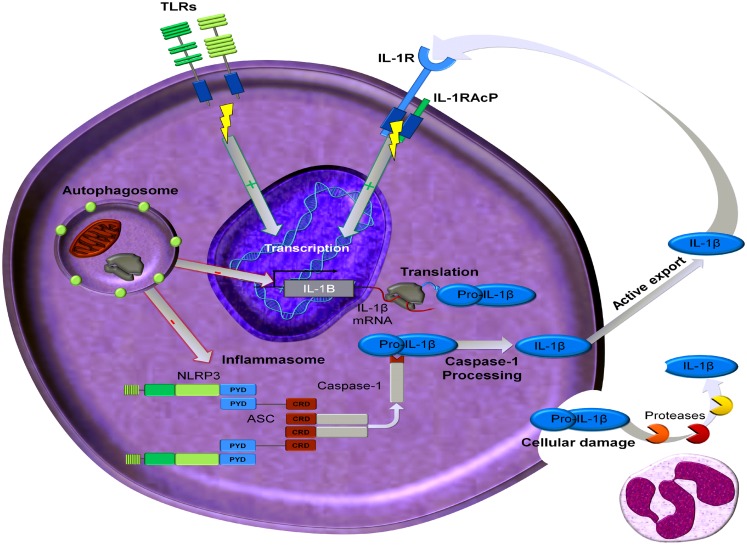
**Processing and regulation of IL-1β**. Transcription of IL-1β mRNA can be induced by ligands that activate Toll like receptors (TLR) or by IL-1 ligands (IL-1α and IL-1β). This transcription can be regulated by autophagy. The precursor of IL-1β, namely pro-IL-1β, can be processed by active caspase-1 which is part of the inflammsome. In addition proteases, predominantly derived from neutrophils, can cleave extracellular pro-IL-1β, which will be present extracellularly in the setting of damaged inflammatory cells. This processed mature and bioactive IL-1β can then induce its own transcription.

IL-1β is a highly inflammatory cytokine as reviewed in Dinarello (2011a), and is primarily a product of monocytes, macrophages, and dendritic cells (DC) as well as B-lymphocytes and NK cells. Caspase-1, an intracellular cysteine protease, is responsible for the conversion of inactive IL-1β precursor into the active cytokine (Figure 2). Caspase-1 likewise needs to be processed in order to become active. This activation of caspase-1 is dependent on a complex of intracellular proteins termed the inflammasome (Agostini et al., 2004; Martinon et al., 2009). One critical component of the inflammasome is NLRP3, also termed cryopyrin since the gene was initially discovered in patients with “familial cold auto-inflammatory syndrome,” a genetic disease characterized by fevers and elevated acute phase proteins following exposure to cold (Hoffman et al., 2001). Human blood monocytes contain constitutively active caspase-1, which is dependent on the presence of the key components of the inflammasome, namely ASC and NLRP3 (Netea et al., 2009). By contrast, other cells, such as macrophages and DC, need an additional trigger to activate caspase-1 (Netea et al., 2009). Non-caspase-1 mechanisms also exist to generate active forms of IL-1β. Sterile inflammation induces fever and increased production of hepatic acute phase proteins, which are absent in mice deficient in IL-1β, but present in mice deficient in caspase-1 (Fantuzzi et al., 1997a; Joosten et al., 2009). This observation can be explained by the fact that proteinase 3 from neutrophils can also process the IL-1β precursor extracellularly into an active cytokine (Coeshott et al., 1999; Joosten et al., 2009), as well as other proteases including elastase, matrix metalloprotease 9, and granzyme A (Figure 2).

Recently, autophagy has been reported to regulate IL-1β production (Saitoh et al., 2008). Autophagy is an ancient process of recycling cellular components, such as cytosolic organelles and protein aggregates, through degradation mediated by lysosomes. Autophagy is activated in conditions of cell stress, hypoxia, starvation, or growth factor deprivation, and it promotes cell survival by generating free metabolites and energy through degradation of the endogenous cellular components (Klionsky, 2007). In addition to its role in the pathophysiology of aging, cancer, and neurodegenerative diseases, autophagy can also modulate inflammatory responses (Schmid and Munz, 2007). A role for autophagy in production of pro-inflammatory cytokines, particularly IL-1β, has emerged with the reported link between ATG16L1 (an autophagy gene) function and IL-1β production. Macrophages from *ATG16L1*-deficient mice produce higher levels of IL-1β and IL-18 after stimulation with lipopolysaccharide (LPS) (Saitoh et al., 2008). These data suggest that higher activation of caspase-1 in *ATG16L1-*deficient mice accounts for the higher production of these caspase-1 dependent cytokines. Indeed, additional studies in *ATG16L1-*deficient mice point toward a regulatory effect of autophagy on caspase-1 activation through modulating the NLRP3 inflammasome (Saitoh et al., 2008; Tschopp and Schroder, 2010; Nakahira et al., 2011; Zhou et al., 2011). Autophagy can thus regulate IL-1β production by influencing caspase-1 activity (Figure 2). Furthermore, autophagosomes present in mouse macrophages can specifically degradate IL-1β precursor (Harris et al., 2011). Moreover, inhibition of autophagy in human primary monocytes leads to increased production of IL-1β (Crisan et al., 2011) (Figure 2). However, in the same cells TNFα production was decreased by autophagy inhibition, suggesting that there are divergent effects of autophagy on the production of these two important pro-inflammatory cytokines. Interestingly, in human cells IL-1β mRNA transcription is elevated when autophagy is inhibited, whereas no effects can be observed on caspase-1 activation (Saitoh et al., 2008; Crisan et al., 2011; Harris et al., 2011). Despite these differences between mouse and human cells, autophagy modulates IL-1β production and inhibition of autophagy increases the production of IL-1β.

The potent inflammatory function of IL-1β is underlined by diseases that are specifically associated with IL-1β production. Although it has been described that IL-1 can play an important role in neurodegenerative diseases such as Alzheimer’s disease, developmental diseases such as schizophrenia (Meyer, 2011) or even stress (Goshen and Yirmiya, 2009) most disease states that have been clearly linked with IL-1β mediated inflammation fall into the category of auto-inflammatory diseases, which are to be distinguished from the classic autoimmune diseases. Although inflammation is common to both auto-inflammatory and autoimmune diseases, in the case of IL-1-mediated disease, there is no evidence for a role of the adaptive immune system in its induction. Persons with activating mutations in one of the key genes that control the activation of caspase-1, namely NLRP3 (cryopyrin), can develop life-threatening systemic inflammation, which can be reversed by blocking IL-1β. Interestingly, in patients with these mutations in NLRP3, there is a decrease in steady state levels of pro-caspase-1 mRNA with IL-1Ra treatment (Goldbach-Mansky et al., 2006), suggesting that IL-1β stimulates its own production and processing. Other studies supporting this concept of IL-1-induced IL-1 have been reported (Goldbach-Mansky et al., 2006; Gattorno et al., 2007; Greten et al., 2007; Boni-Schnetzler et al., 2008). This explains the auto-inflammatory nature of these IL-1 mediated diseases, namely an initial trigger induces the production of IL-1β, which thereafter can induce itself. Type 2 diabetes appears to be an example of an auto-inflammatory disease where glucose induces IL-1β production from the insulin-producing beta cell and IL-1β induces the beta cell to produce its own IL-1β (Maedler et al., 2002).

No spontaneous disease has been reported in mice deficient in IL-1β. However, upon challenge, IL-1β-deficient mice differ significantly in their responses from wild-type mice. The most dramatic is the response to local inflammation induced by a local irritant. IL-1β-deficient mice will not have an acute phase response, develop anorexia, and have no fever within the first 24 h (Zheng et al., 1995; Fantuzzi et al., 1997b). These effects have also been observed in the same model using anti-IL-1R type I antibodies in wild-type mice (Zheng et al., 1995; Fantuzzi et al., 1997b). Reduced inflammation has also been observed in IL-1β-deficient mice that are exposed to zymosan-induced peritonitis (Fantuzzi et al., 1997b). IL-1β-deficient mice injected with LPS have little or no expression of leptin mRNA or leptin protein (Faggioni et al., 1998), however they do have elevated febrile responses to LPS, IL-1β, or IL-1α when compared to wild-type mice (Fantuzzi et al., 1996).

Interleukin-1β enhances T-cell activation and recognition of antigen. The specificity of this response was however not known initially. IL-1β, together with IL-6, and TGFβ have been reported to induce the development of Th17 cells, while IL-23 has been reported to be important for the maintenance of Th17 cells (Weaver et al., 2006; Dong, 2008; van de Veerdonk et al., 2009). The combination of IL-23 and IL-1β induces the development of human Th17 cells (Wilson et al., 2007). Interestingly, these cells also released IFNγ, displaying a phenotype common to both Th17 and Th1 cells (Wilson et al., 2007). The strong capacity of IL-1 to induce Th17 differentiation has also been linked to the induction and release of prostaglandins (Dinarello, 2011b). PGE2 are inducers of Th17 induction and inhibitors of cyclooxygenase decrease IL-17 production (Chizzolini et al., 2008; Smeekens et al., 2010). Furthermore, engagement of the aryl hydrocarbon receptor, a pathway demonstrated to be crucial for the generation of Th17 cells, has been shown to strongly induce IL-1β (Henley et al., 2004). IL-1β is also required for the production of IL-17 by NKT cells (Moreira et al., 2011) and of IL-22 from NK cells (Hughes et al., 2010).

Cytokines belonging to the IL-1 family have also been described to modulate neurons and functions of the central nervous system (CNS). For example IL-1β and its antagonist IL-1Ra have been extensively described for their ability to act within the CNS as modulators of hippocampal memory, as well as involvement in neuronal death (Yirmiya and Goshen, 2011; Hanamsagar et al., 2012). From these studies it is clear that in some context these cytokines that belong to the IL-1 family not only exert a pathological role but also play a role in homeostasis. These emerging observations underscore that the functions of inflammatory cytokines such a IL-1β are not only confined to the classical inflammatory response.

### Interleukin-33

IL-33 belongs to the IL-1 subfamily, and was formerly termed IL-1F11. IL-33Rα is the ligand binding chain for IL-33 (Schmitz et al., 2005), and the co-receptor for IL-33 is the IL-1RAcP, which is also the co-receptor for IL-1α and IL-1β. The IL-33Rα chain is similar to IL-1R1, since it can bind the ligand but requires the IL-1RAcP to signal (Ali et al., 2007; Chackerian et al., 2007). The structure of IL-33 is closer to IL-18 than to IL-1β. Initially, IL-33 was considered closely related to IL-1β and IL-18 because the IL-33 precursor contains a caspase-1 site (Schmitz et al., 2005). However studies have revealed that caspase-1 cleaving of IL-33 actually results in loss of IL-33 activity and that the full-length IL-33 precursor can bind to IL-33Rα and is active (Cayrol and Girard, 2009). In addition, it has been reported that the caspase-1 cleavage site is similar to the consensus sequence for caspase-3 and that intracellular IL-33 precursor is a substrate for caspase-3 (Cayrol and Girard, 2009). Precursor IL-33 can also be processed by neutrophil proteinase 3 (PR3) into a biological active form of IL-33, however increasing the incubation time of PR3 will decrease the biological activity of IL-33 (Bae et al., 2012). Next to PR3 cleavage, neutrophil elastase and cathepsin G can cleave the IL-33 precursor, which results in the generation of IL-33 with different N-termini and varying levels of activity (Lefrancais et al., 2012). Thus, extracellular IL-33 is released as a precursor and can be processed by neutrophil enzymes which will generates active forms with varying levels of activity.

The dominant biological activity of IL-33 is the induction of Th2 cytokines, IL-4, IL-5, and IL-13 as well as other properties anticipated for a Th2 type cytokine. Therefore, the role of IL-33 in lung inflammation such as allergic type asthma has been studied extensively. Administration of IL-33 into the airways triggers an immediate allergic response in the lung of naïve mice and worsens the response in mice sensitized to antigen (Louten et al., 2011). When human IL-33 is administered to mice eosinophilic infiltration is a prominent finding in the lung and in allergic rhinitis as well as allergic conjunctivitis (Matsuba-Kitamura et al., 2010). Interestingly, it was recently described that interleukin-1α can control allergic sensitization to inhaled house dust mite (HDM) via the epithelial release of IL-33 (Willart et al., 2012). Mice deficient in IL-33Rα do not develop a Th2 response to Schistosoma egg antigen, and mice deficient in IL-33, are highly susceptible to *Strongyloides venezuelensis* (Yasuda et al., 2012). This infection induces a unique class of cells called natural helper cells or nuocytes, which produce IL-5 and IL-13 upon activation by IL-33, which results in eosinophilic infiltration into the lungs. This pulmonary eosinophilic inflammation causes damage that is IL-33 and IL-5 dependent (Yasuda et al., 2012).

Other impressive pathological findings such as changes in the arterial walls and intestinal tissues have also been observed when human IL-33 is injected in mice (Schmitz et al., 2005; Kim et al., 2012). In mice deficient in IL-33Rα, there is myocardial hypertrophy, ventricle dilation, and fibrosis of the heart suggesting that IL-33 plays a protective role in the heart (Sanada et al., 2007). Moreover, elevated levels of the extracellular domain of IL-33Rα predict outcomes in patients that have had a myocardial infarction (Sanada et al., 2007). Furthermore, administration of recombinant IL-33 inhibits the phosphorylation of IκB and reduces hypertrophy and fibrosis in a model of cardiomyocyte hypertrophy (Sanada et al., 2007). One of the more challenging aspects is the role of the IL-33 signaling pathway in the ApoE deficient mouse model of atherosclerosis. Arterial wall plaques of ApoE deficient mice on a high-fat diet contain IL-33 and IL-33Rα. Atherosclerotic plaques were markedly reduced when these mice were treated with IL-33, however when Insoluble IL-33Rα was administered to neutralize IL-33 signaling the disease worsened (Miller et al., 2008).

Clearly IL-33 has properties that go beyond its role of inducing Th2 responses. For example, IL-33 can induce potent CD8(+) T-cell (CTL) responses to replicating, prototypic RNA, and DNA viruses in mice (Bonilla et al., 2012). Moreover, IL-33 is identical to a nuclear factor which is dominantly expressed in high endothelial venules (HEV) called NF-HEV (Carriere et al., 2007). Constitutive nuclear localization of IL-33 has also been reported in several other cell types such as type II lung epithelial cells (Yasuda et al., 2012), epithelial cells (Moussion et al., 2008), and pancreatic stellate cells (Masamune et al., 2010). IL-33 binding to DNA and acting as a nuclear factor resembles closely the IL-1α binding to chromatin and IL-1α functioning as a nuclear factor (Stevenson et al., 1997; Werman et al., 2004; Cohen et al., 2010). IL-33 precursor can bind NF-κB p65 and IL-1β-induced TNFα is reduced in cells overexpressing the IL-33 precursor (Ali et al., 2011). These data suggest that next to the ability of IL-33 to induce T-cell responses, IL-33 possesses anti-inflammatory activity which appears to be dependent on nuclear sequestration (Cohen et al., 2010).

## The IL-18 and IL-37 Subfamily

### Interleukin-18

Interleukin-18 is extensively reviewed in this issue of Frontiers in Immunology by Dr. C. Dinarello.

### Interleukin-37

IL-37, formerly termed IL-1F7, lacks a signal peptide and has a caspase-1 site. IL-37 can translocate to the nucleus following stimulation, similar to IL-1α and IL-33 (Sharma et al., 2008). Inhibition of caspase-1 markedly reduces nuclear entry of IL-37 (Sharma et al., 2008), suggesting that IL-37 translocates to the nucleus after caspase-1 processing, and acts as a transcriptional modulator reducing the production of LPS-stimulated pro-inflammatory cytokines. It must be noted that the secretion of IL-37 has not been documented with any certainty. It is likely that the IL-37 precursor exits the cell during cell death and that this precursor suppresses LPS-induced IL-1β, IL-6, and TNFα (Nold et al., 2010). It was from the first reports on IL-37 that recombinant IL-37 bound to the IL-18Rα (Pan et al., 2001; Kumar et al., 2002). In IL-37 transgenic mice this binding of IL-37 to IL-18Rα has also been observed (Nold et al., 2011), and it has been reported that IL-37 specifically binds to the third domain of the IL-18Rα (Bufler et al., 2002). However, IL-37 does not act as a classical receptor antagonist for IL-18, despite these studies showing binding of IL-37 to the IL-18Rα chain. High concentrations of IL-37 do not inhibit recombinant IL-18-induced IFNγ, and recombinant IL-37 modestly reduces IL-18-induced IFNγ in the presence of low concentrations of IL-18BP (Bufler et al., 2002).

A mouse homolog for human IL-37 has not been identified, therefore a strain of transgenic mice has been generated to study the *in vivo* biological function of IL-37 (Nold et al., 2010). No obvious phenotype in homozygous IL-37 transgenic mice (IL-37 tg) mice has been observed and breed normally. Importantly, these mice do not constitutively express mRNA levels of IL-37, which is most likely due to a functional instability sequence found in IL-37 that limits the half-life of IL-37 mRNA (Bufler et al., 2004). Upon stimulation with IL-1β or LPS, expression of IL-37 increases after 4–24 h and the IL-37 precursor can be found in peripheral blood cells isolated from the transgenic mice (Nold et al., 2003). Compared to wild-type mice, IL-37 transgenic mice are protected against LPS challenge (Nold et al., 2010). They display significantly less hypothermia, acidosis, hyperkalemia, hepatitis, and dehydration during LPS challenge. In addition, IL-6 and TNFα production is significantly less in whole blood cultures from IL-37 transgenic mice when stimulated by IL-1β or the combination of IL-12 plus IL-18. This anti-inflammatory activity of IL-37 is not limited to a reduction of the cytokines and chemokines, also DC isolated from the spleen of IL-37 transgenic mice have a marked reduction in their expression of CD86 and MHC II after LPS challenge (Nold et al., 2010). IL-37 transgenic mice subjected to dextran sulfate sodium (DSS)-induced colitis have significantly less clinical disease compared to wild-type mice (McNamee et al., 2011). A decreased leukocyte recruitment into the colonic lamina propria was observed in IL-37Tg mice which was associated with decreased proinflammatory cytokine production. Wild-type mice reconstituted with bone marrow from IL-37 transgenic mice were protected from colitis, suggesting that IL-37 originating from hematopoietic cells is sufficient to exert protective anti-inflammatory effects.

## The IL-36 Subfamily

The IL-1 family members IL-1F5, IL-1F6, IL-1F8, IL-1F9, and IL-1F10 are now termed IL-36Ra, IL-36α, IL-36β, IL-36γ, and IL-38 respectively (Dinarello et al., 2010). Each member of the IL-36 subfamily binds to the IL-1Rpr2, now termed IL-36R (Towne et al., 2004). The IL-36 subfamily is closely related to the IL-1 subfamily because similar to the IL-1α and IL-1β and IL-33, the IL-36R forms a signaling complex with the IL-1RAcP (Towne et al., 2004; Ali et al., 2007).

### Interleukin-36α, β, γ (IL-36)

IL-36α, IL-36β, and IL-36γ all have agonistic characteristics and signal through the IL-36R (Towne et al., 2004; Magne et al., 2006; Chustz et al., 2011). These IL-36 cytokines are mainly expressed in keratinocytes, bronchial epithelium, brain tissue, and monocytes/macrophages (Smith et al., 2000; Barksby et al., 2009). LPS derived from *E. coli* or *P. gingivalis* specifically induces expression of IL-36γ, but not IL-36α or IL-36β in THP-1 cells (Barksby et al., 2009). Peripheral blood lymphocytes are able to express IL-36γ in response to α-particles, which can be used for targeted cancer therapy (Turtoi et al., 2010), and T-lymphocytes have been reported to express IL-36α and IL-36β (Smith et al., 2000; Li et al., 2010; Vigne et al., 2011). Interestingly, it has recently been shown that γδ T-cells can express IL-36β under specific conditions (Yang et al., 2010). IL-36 cytokines like IL-1 and IL-18 also need to be processed in order to gain full bioactivity, although the enzyme responsible still remains to be determined (Towne et al., 2011).

IL-36β can induce expression of itself, and thus an autocrine/paracrine loop similar to IL-1 also seems to be present in the IL-36 subfamily of cytokines (Dinarello et al., 1987; Carrier et al., 2011). IL-36α, IL-36β, and IL-36γ can induce IL-17 and TNF expression in keratinocytes, which can be synergized by the cytokine IL-22 (Carrier et al., 2011). Furthermore, several reports indicate that epidermal growth factor signaling regulates the expression of IL-36α and IL-36β in the skin (Yang et al., 2010; Franzke et al., 2012) suggesting an important role of the agonists IL-36α and IL-36β in skin homeostasis. In line with this is the observation that transgenic mice which overexpress the IL-36α gene in basal keratinocytes display acanthosis and hyperkeratosis of the skin, which are characteristics of psoriatic skin lesions (Blumberg et al., 2007). IL-36 cytokine expression in bronchial epithelial cells can be induced by several pro-inflammatory stimuli (Vos et al., 2005; Chustz et al., 2011). In human lung fibroblasts, IL-36γ induces the chemokine IL-8 and the Th17 chemokine CCL20 (Chustz et al., 2011), suggesting that IL-36 cytokines can contribute to pro-inflammatory responses and in particular neutrophilic airway inflammation.

Furthermore, the IL-36R is highly expressed on microglial cells and astrocytes (Lovenberg et al., 1996; Berglöf et al., 2003; Wang et al., 2005). Murine IL-36β is expressed in neuron cells and in glial cells, but cannot be upregulated by LPS or IL-1β stimulation (Berglöf et al., 2003; Wang et al., 2005). Intraventricular injection of recombinant non-processed mouse IL-36β does not induce any of the classical IL-1 like responses such as fever or modification of food intake and body weight in mice (Berglöf et al., 2003). However, it must be noted that these studies have been performed with non-processed IL-36 agonists, which is shown to have significantly less bioactivity compared to its processed form (Towne et al., 2011).

### IL-36 receptor antagonist

IL-36Ra shares homology with IL-1Ra but is unable to bind to the IL-1R1 since it significantly differs in loop conformations from IL-1Ra (Dunn et al., 2003). IL-36Ra inhibits IL-36γ-induced NFκB activation (Debets et al., 2001; Towne et al., 2004) in a way similar to IL-1Ra (Towne et al., 2011). However unlike IL-1Ra, IL-36Ra needs to be processed in order to gain antagonistic properties (Towne et al., 2011). Interestingly, IL-36Ra itself can induce mRNA of IL-4 and protein expression in glia cells, which can be attenuated by anti-SIGIRR antibodies. Moreover, the anti-inflammatory action IL-36Ra *in vivo* in the brain is dependent on IL-4 and SIGIRR (Costelloe et al., 2008). IL-36Ra reduces fungal-induced Th17 responses, however not in a classical dose-dependent manner (van de Veerdonk et al., 2012; Gresnigt et al., 2013). These reports suggest that IL-36Ra might be able to recruit the anti-inflammatory IL-1 orphan receptor SIGIRR and activate an anti-inflammatory signaling pathway, and thus does not act as a classical receptor antagonist such as IL-1Ra.

The importance of the biological activity of IL-36Ra in regulating skin inflammation has been demonstrated by several reports. IL-36Ra deficiency exacerbates skin lesions in IL-36α transgenic mice (Blumberg et al., 2007). Phorbol ester treatment of mouse skin overexpressing IL-36α results in an inflammatory condition with macroscopic and histological similarities to human psoriasis (Blumberg et al., 2010), and characteristic inflammation of human psoriatic skin transplanted into immunodeficient mice is dependent on the IL-36R (Blumberg et al., 2010). In patients with psoriasis anti-TNF treatment results in decreased expression of the IL-36 agonists and IL-36Ra, which was associated with improved clinical outcome (Johnston et al., 2011). This increased expression of *IL-36* agonists correlates with Th17 cytokines in human psoriatic skin lesions, although the expression of IL-36Ra by IL-17-stimulated keratinocytes derived from patients with psoriasis does not differ from healthy controls (Carrier et al., 2011; Muhr et al., 2011). Moreover it has recently been shown that mutations in *IL-36RN* can cause a rare life-threatening disease called general pustular psoriasis (GPP) (Marrakchi et al., 2011; Onoufriadis et al., 2011; Sugiura et al., 2012; Farooq et al., 2013). The currently found mutations in *IL-36RN* lead to introduction of a premature stop-codon, frameshift mutation, or an amino acid substitution which were found to result in a misfolded IL-36Ra protein that is less stable and poorly expressed (Marrakchi et al., 2011; Sugiura et al., 2012; Farooq et al., 2013). The misfolded IL-36Ra has less affinity with the IL-36R compared to the wild-type IL-36Ra protein, and therefore is not able to dampen IL-36R-mediated inflammation (Marrakchi et al., 2011; Sugiura et al., 2012; Farooq et al., 2013). These data indicate that IL-36Ra is a receptor antagonist, and that IL-36 signaling plays a significant role in regulating skin inflammation.

IL-36 cytokines might play a significant role in joint disease. Remarkably, only IL-36β is expressed joints of mice and humans (Magne et al., 2006). Interestingly, IL-36β can be measured in the serum of healthy human volunteers, but when serum IL-36β concentrations of healthy volunteers are compared to serum concentrations in rheumatoid arthritis there were no significant differences observed (Magne et al., 2006). However, a recent study showed that the IL-36R was not involved in the inflammatory response in a mouse model of collagen induced arthritis (Lamacchia et al., 2013). In a Caucasian cohort polymorphisms in IL-36β have been associated with spondylitis ankylopoetica, but not this association was not observed in Asian cohorts (Wu and Gu, 2007; Kim et al., 2008).

IL-36γ expression in the lung is significantly increased compared to non-challenged mice in a murine model of HDM-induced allergic inflammation. When recombinant IL-36γ is given intratracheally, this will result in neutrophil influx, but not eosinophilic influx in the lungs of mice, suggesting that IL-36γ is more involved in the regulation of neutrophilic airway inflammation (Chustz et al., 2011; Ramadas et al., 2011, 2012). Bronchial epithelial cells from patients with asthma that were infected with rhinovirus show a higher expression of IL-36γ compared to infected cells from healthy controls (Bochkov et al., 2010). These data support the concept that IL-36 cytokines might also play a significant role in regulating airway inflammation.

### Interleukin-38

IL-1F10 has recently been renamed IL-38 (Dinarello et al., 2010). IL-38 shares 43% homology with IL-36Ra and 41% homology with IL-1Ra (Bensen et al., 2001). The IL-38 precursor lacks a signal peptide and is 152 amino acids in length, and the natural N-terminus is unknown (Bensen et al., 2001). There is no caspase-1 consensus cleavage site present in the precursor of IL-38. IL-38 is predominantly expressed in the skin and in proliferating B-cells of the tonsil (Lin et al., 2001). The allele combinations that include IL-38 polymorphisms are associated with psoriatic arthritis and ankylosing spondylitis (Chou et al., 2006; Rahman et al., 2006; Guo et al., 2010), suggesting that IL-38 plays a role in the pathogenesis of these inflammatory diseases. Moreover, and suggesting an important role for this cytokine in human cardiovascular disease, polymorphisms in IL-38 were associated with CRP concentrations in humans in addition to polymorphisms in CRP, IL-6 receptor, and NLRP3 that were also associated with CRP concentrations (Dehghan et al., 2011).

Although it has been reported earlier that IL-38 binds to the IL-1 receptor type I this binding affinity of recombinant IL-38 was low (Lin et al., 2001), and more recently it was demonstrated that IL-38 can bind to the IL-36R similar to IL-36Ra (van de Veerdonk et al., 2012). The only biological activity reported so far is that IL-38 can reduce *Candida*-induced T helper 17 responses (van de Veerdonk et al., 2012). Notably, the dose-response suppression of IL-38 as well as that of IL-36Ra of *Candida*-induced IL-22 and IL-17 was not similar to the classic dose-response of IL-1 receptor antagonist, because low concentrations were optimal for inhibiting IL-22 production (van de Veerdonk et al., 2012). A non-classical dose-response has now been observed for IL-36Ra, IL-37, and IL-38 activity and it remains to be determined what the underlying mechanism and biological significance is of these findings.

## Interleukin-1 Receptor Antagonist

The IL-1 receptor is expressed in nearly all tissues and its antagonism prevents receptor binding of either IL-1α or IL-1β, therefore its biological function is as diverse as the roles of IL-1α and IL-1β apart and combined. IL-1Ra can inhibit these responses by binding to the IL-1R1 and preventing the recruitment of IL-1RAcP, which will block IL-1 signaling (Dinarello, 1996). The potent inhibitory effect of IL-1Ra and its importance as a regulating protein in IL-1-mediated inflammation is underlined by a disease called deficiency in interleukin-1 receptor antagonist (DIRA) (Aksentijevich et al., 2009). This disease is characterized by severe sterile multifocal osteomyelitis, periostitis, and pustulosis (Aksentijevich et al., 2009). The life-threatening overwhelming inflammation of skin and bones in these patients can be resolved by treatment with recombinant IL-1Ra. Next to treating this rare disease it should be highlighted that IL-1Ra as a recombinant molecule is successful and on the rise as a new therapeutic agent for many diseases. The use of blocking IL-1 is extensively reviewed in Dinarello et al. (2012), and treating auto-inflammatory diseases with IL-1Ra such as Muckle-wells or gout is highly effective, and a growing list of diseases in which blocking IL-1 signaling with IL-1Ra is growing (Dinarello et al., 2012).

## Conclusion

It is becoming clear that most members of the IL-1 family primarily promote inflammation and enhance specific acquired immune responses, while some members can provide a brake on inflammation, such as IL-1Ra and IL-36Ra. We are just beginning to understand the biological function of the new IL-1 family members, IL-37 and the cytokines belonging to the IL-36 subfamily, and we are increasingly appreciating the potency of blocking IL-1 in disease. This underscores that long after the initial discovery of IL-1, the cytokine biology of the IL-1 family is still contributing to understanding pathology of disease and remains an exciting field to study.

## Conflict of Interest Statement

The authors declare that the research was conducted in the absence of any commercial or financial relationships that could be construed as a potential conflict of interest.
